# A Meta-Analysis to Determine the State of Biological Control of Aphanomyces Root Rot

**DOI:** 10.3389/fmolb.2021.777042

**Published:** 2022-02-02

**Authors:** Ashebir T. Godebo, Naomi Marie J. Wee, Christopher K. Yost, Fran L. Walley, James J. Germida

**Affiliations:** ^1^ Department of Soil Science, University of Saskatchewan, Saskatoon, SK, Canada; ^2^ Department of Biology, University of Regina, Regina, SK, Canada; ^3^ Institute for Microbial Systems and Society, University of Regina, Regina, SK, Canada

**Keywords:** *Aphanomyces euteiches*, aphanomyces root rot, biocontrol, meta-analysis, microbial biocontrol agents

## Abstract

The increasing incidence and prevalence of the pathogen *Aphanomyces euteiches* in various pulse-growing regions worldwide necessitates the development of effective management strategies, including biological control agents. Numerous labs have undertaken research examining biological control methods to evaluate aphanomyces root rot suppression in multistep processes that include isolation of inhibitory organisms, lab assays, growth chamber assays, and field trials. Given the emergence of various biocontrol agents and the need to mitigate aphanomyces yield losses, we have undertaken a meta-analysis approach to analyze the effectiveness of biocontrol agents in relation to application method, biocontrol agent richness, biocontrol agent type, the type of study, and reporting system-oriented moderator variables. An effect size, calculated as a natural log response ratio, resulted in a summary weighted mean of −0.411, suggesting the overall effectiveness of biocontrol agents (*p* < .001). Aphanomyces root rot suppression using biological treatments showed significant heterogeneity for all moderator variables, confirming that the studies do not share a common effect size and the use of a random effect model was appropriate. Across studies, meta-analyses revealed that soil amendments, biocontrol agent application as a seed coating and suspension, bacterial and fungal biocontrol agents, mixed applications, growth chamber and field studies, and qualitative and quantitative reporting systems were all associated with significantly positive outcomes for aphanomyces root rot suppression. Our findings suggest that there is potential promise for biological control of aphanomyces root rot, and more field trials need to be conducted to demonstrate the efficacy level observed under growth chamber conditions. Moreover, we identified a lack of detailed understanding of the mechanism(s) of biological control of aphanomyces root rot as a research priority.

## 1 Introduction

Aphanomyces root rot caused by the soil-borne oomycete pathogen *Aphanomyces euteiches* affects the belowground portion of the developing plant, leading to poor yields in pulse crops (Saskatchewan Pulse Grower, 2017). Although *A. euteiches* can be isolated from alfalfa (*Medicago sativa* L.), snap bean (*Phaseolus vulgaris* L.), red kidney bean (*Proteus vulgaris* L.), faba bean (*Vicia faba* L.), red clover (*Trifolium pratense* L.), white clover (*Trifolium repens* L.), and several weed species, it causes the greatest economic impacts to pea (*Pisum sativum* L.) and lentil (*Lens culinaris* Medik.) crops (Wu et al., 2018). The genus *Aphanomyces* comprises three subgroups: *Aphanomyces* plant pathogens, *Aphanomyces* aquatic animal pathogens, and *Aphanomyces* saprophytic species (Gaulin et al., 2018). Among the plant pathogens, *A. euteiches* is the most destructive pathogen (Wu et al., 2018). Moreover, although the oomycete pathogen *Pythium* can be controlled by seed treatments (Chatterton et al., 2016), and fungicides that control root rot–causing pathogens such as *Fusarium* are available, they are not providing adequate protection against aphanomyces root rot (Top Crop Manager, 2021).

Currently, no successful management method exists for control of aphanomyces root rot. Crop rotation, disease avoidance, and host resistance are reported to offer limited success (Wakelin et al., 2002; Sauvage et al., 2007; Hughes and Grau, 2013; Conner et al., 2013). At present, INTEGO™ Solo (ethaboxam) and Vibrance^®^ Maxx RFC are two registered fungicides for suppressing early season aphanomyces root rot in field pea and lentil in Canada (Guide to Crop Protection, 2021). Due to the lack of effective control methods and the growing demand for sustainable production practices, biological control methods are proposed to offer an effective, safe, and environmentally favorable alternative (Godebo et al., 2020). Xue, 2003 and Godebo et al. (2020) report that biocontrol of aphanomyces root rot could be achieved using antagonistic microbes. Several biocontrol agents with varying levels of biocontrol efficacy are commercialized as biocontrol agents for other plant pathogens. Some examples of these products include Integral (*B. subtilis* MBI 600) for *Rhizoctonia* spp. and *Fusarium* spp.; Kodiak (*Bacillus subtilis* GB03) for *Rhizoctonia* spp., *Fusarium* spp., and *Aspergillus* spp.; and Serenade (*B. subtilis* QST 713) for *Botrytis* spp., *Sclerotinia* spp., *Xanthomonas* spp., and *Erwinia* spp. (Liu et al., 2017). The nomenclature of these *Bacillus subtilis* species is now changed to *Bacillus velezensis* (Dunlap et al., 2016).

Several factors, including the type of biocontrol agent, biocontrol agent richness, method of application, and study type, influence the effectiveness of a biocontrol agent in controlling plant pathogens (Chandrasekaran et al., 2016). For instance, mixed application of biocontrol agents may be assumed to offer more significant suppression of plant pathogens. However, a study by Dandurand and Knudsen (1993) reports that aphanomyces root rot suppression was not significantly different when pea seeds were treated with a combination of *Trichoderma harzianum* and *Pseudomonas fluorescens* strains compared with treatment with *T. harzianum* alone. Thus, due to these and other factors, biocontrol research reports indicate the level of plant disease suppression is inconsistent among and between studies investigating similar biocontrol agents. Moreover, an increasing number of studies report that several biocontrol agents demonstrate variable efficacy in controlling aphanomyces root rot. Because of these apparently contradictory observations, there is a need to conduct a meta-analysis study to determine the state of the biological control of aphanomyces root rot and draw conclusions that will direct future research.

Meta-analysis provides a critical and quantitative review of research data extracted from various studies to determine the influence of treatment (experimental) factors on effect sizes and evaluate possible publication bias (Rosenberg et al., 2004). Meta-analysis offers the opportunity to draw a holistic conclusion based on primary experimental findings from several studies (Nelson et al., 2015). Others use this approach to examine biocontrol agents. For example, Chandrasekaran et al. (2016) performed a meta-analysis to quantify the overall efficacy of biocontrol agents in reducing *Ralstonia* wilt and their effect on growth promotion and crop yield. Shrestha et al. (2016) conducted a meta-analysis to understand the efficacy of the nonchemical practice of anaerobic soil disinfestation on a range of soil-borne pathogens, nematodes, and weeds. In this review, meta-analysis was used to quantitatively analyze the findings of 24 published studies (Supplementary Table S1) on the biological control of aphanomyces root rot.

## 2 Materials and Methods

### 2.1 Literature Review and Data Collection

A database of research articles investigating the potential for biological control of *A. euteiches* was assembled using Web of Science (https://www.webofscience.com) in April 2021. The keyword used for the initial search, “*Aphanomyces*,” returned 1285 search results. These search results were filtered to 449 articles using the keyword “*Aphanomyces euteiches*” and to 41 using the keyword “biological control.” Articles were also searched in other sources, namely Science, (https://www.sciencemag.org), Nature (https://www.nature.com), Elsevier-Science Direct (https://www.sciencedirect.com), Springer (https://www.springer.com), Wiley & Sons (https://onlinelibrary.wiley.com), Scopus (https://www.scopus.com), and Google Scholar (https://scholar.google.com). The articles were further screened for meeting inclusion criteria. The criteria for including a study were 1) a treatment with at least one biocontrol agent, 2) reporting a measure of disease intensity (disease incidence or disease severity), and 3) employing statistical analysis between treatments. This meta-analysis did not include studies solely investigating chemical control agents. Based on these inclusion criteria, the meta-analysis included 24 published articles spanning 1990 to 2020 (Supplementary Figure S1 and Supplementary Table S1).

Treatment means and sample sizes were collected for each study to investigate the impact of biocontrol agents on suppressing aphanomyces root rot in relation to five factors determined as moderator variables. Biocontrol agent treatment means were those that were evaluated for biocontrol activities in the respective studies. In contrast, positive controls that only received the pathogen were considered control means (i.e., positive controls in each study). Multiple biocontrol agents studied in an article were treated as independent studies commonly regarded as paired observations in meta-analysis literature. Hence, each biocontrol agent represented individual units in this meta-analysis. For instance, Godebo et al. (2020) report biocontrol efficacy data for 20 strains from two growth chamber studies. Each trial evaluated 10 different bacteria; accordingly, that article resulted in 20 studies, and this approach is consistent with that used by Shrestha et al. (2016). Thus, the entire data set consisted of 162 “studies” from 24 published articles (Supplementary Table S1 and Supplementary Table S2).

### 2.2 Moderator Variable and Categorical Analysis

A moderator variable is a variable that can alter the association between the study factors (independent variables: for example, biocontrol agent application method) and the outcome (the dependent variables: for example, response ratio) (Allen 2017). Various moderator variables affecting aphanomyces root rot suppression were identified and categorized and categorical moderator analysis was conducted using Comprehensive Meta-Analysis Version 3 software (Borenstein et al., 2013). The first moderator variable was the application method, and it was categorized into three levels: seed coating, suspension, and amendment. Seed coating and suspension refer to biocontrol inoculants applied via seed coating and liquid formulations, respectively. The amendment describes plant growing media amended with compost, green manure, and other plant products for the purpose of suppressing aphanomyces root rot. The second variable was biocontrol agent richness, and it describes the number of biocontrol agents inoculated for the biocontrol assessment of *A. euteiches* in a single inoculation. It was evaluated into two levels as single and mixed organism inoculation. Single organism inoculation refers to the inoculation of a single biocontrol agent. In contrast, mixed organism inoculation is the application of two or more biocontrol agents as a coinoculation. The third variable was biocontrol agent type, which represents studies that report the taxonomical identity and type of the biocontrol agents. This was divided into five levels: bacteria, fungi, green manure, compost, and plant product that did not incorporate synthetic chemical products. The fourth variable was the type of the study, which denotes the studies that investigated the inhibition of *A. euteiches* growth and suppression of root rot symptom development. This was categorized into three levels: lab, growth chamber, and field studies. The lab study refers to studies conducted under laboratory conditions, including culture media–based *A. euteiches* growth inhibition and other inhibition assays undertaken in laboratory settings. The last moderator variable was the pathogen suppression reporting system analyzed in two groups as qualitative or quantitative. The qualitative reporting system represented experimental data collected using a disease rating scale. In contrast, the quantitative reporting system included experimental data that measured pathogen infestation levels, for example, quantifying *A. euteiches* level in roots and measuring oogonia (*A. euteiches* developmental stage) production and plant dry weight. We did not investigate the host plant species (example: field pea versus lentil) and mechanism of action as moderator variables due to the lack of sufficient data representing these two variables.

### 2.3 Effect Size Calculation and Meta-Analysis

The effect size of investigated biocontrol agents was estimated as a natural log of the response ratio (*lnR*) as a measuring standard to assess the effectiveness of the treatments covered in each study. A response ratio is the ratio of measured outcome in the treated (treatment) group relative to measured outcomes in the treatments that received the pathogen only (i.e., positive controls in each study) as stated by Rosenberg et al. (2000). This meta-analysis used a random-effect model that assumes true effects vary across studies instead of a fixed model that considers the same value for all studies. Therefore, the effect size for each study was calculated according to the following formula (Chandrasekaran et al., 2016):

$$\mathrm{lnR = ln}\left(X_{t}/X_{c}\right)\mathrm{= ln}\left(X_{t}\right)\mathrm{-ln}\left(X_{c}\right).$$

*R* is the response ratio, ***X***_*t*_ is the biocontrol agent treatment mean, and ***X***_*c*_ is the control mean. Because the majority of the studies did not report a measure of dispersion, a nonparametric variance was calculated as stated in Shrestha et al. (2016) as

$$\mathrm{VlnR=}\left(n_{t}+n_{c}\right)/\left(n_{t}*n_{c}\right).$$

*V*_*lnR*_ *is* the natural log of the response ratio variance, ***n***_*t*_ is the treatment mean sample size, whereas ***n***_*c*_ is the sample size of the control mean.

### 2.4 Test of Heterogeneity

During the moderator variable analysis, three **Q** statistics were generated per factor, a measure of weighted squared deviation used to assess heterogeneity. The first **Q** was the variation within categories (**Q**_**w**_), the second was the variation between categories (**Q**_**b**_), and the last was the total heterogeneity (**Q**_**t**_), which is composed of the within- and between-study variation. In addition, heterogeneity was measured using a descriptive index designated as ***I*** ^2^, and it measures the ratio of true variation (heterogeneity) to total variation across studies as described by Shrestha et al. (2016):

$$I^{2}=\left(Q_{t}\;-\;\mathrm{Df}\right)/Q_{t}*\mathrm{100}\%,$$

where **Df** represents the expected variation **Q**_**w**_ and **Q**_**t**_ **–Df** denotes the excess variation (**Q**_**b**_). When **Df** is larger than **Q**_**t**_ **, *I*** ^2^ is set to zero, and a value of zero means no true heterogeneity. A positive value indicates true heterogeneity, and large values represent a more significant proportion of the observed variation due to true heterogeneity among studies. Thus, much of the total heterogeneity can be addressed by subdividing studies into groups of interest. Homogeneity was considered invalid when the *p*-value for the Q-test (*P*_hetero_) for heterogeneity was less than .1 (Bristow et al., 2013; Iacovelli et al., 2014; Shrestha et al., 2016).

### 2.5 Publication Bias

Because meta-analysis is accepted as comprehensive, publication bias (i.e., systematic unrepresentativeness) is often raised as a concern with such analyses. This is due to the trend that significant treatment differences get published more than nonsignificant findings. Although it is difficult to find direct evidence of publication bias, it can be estimated using a statistical approach (Madden and Paul, 2011; Koricheva and Gurevitch, 2014). The concept behind publication bias analysis is that studies with smaller sample sizes and/or higher variance usually have greater effect sizes than large studies and have much more precision. Therefore, the publication bias analysis method involves understanding the relationship between study effect size and precision. Our meta-analysis publication bias was investigated statistically with Egger’s regression test (Egger et al., 1997). The analysis output is presented graphically with funnel plots of effect sizes versus precision (standard error^−1^). In addition, the iterative trim and fill method was used to visualize how the summary effect size shifts when significant bias is discarded.

## 3 Results

### 3.1 Measure of Efficacy

**FIGURE 1:**
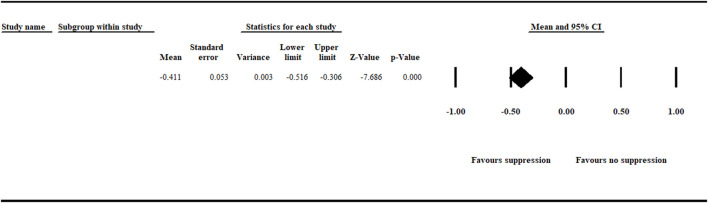
Cumulative analysis of effect size measuring efficacy on biological control of aphanomyces root rot. The analysis detected a significant (*p* < .001) negative summary effect size [−0.411 (CI −0.516 to −0.306)] suggesting suppression of *A. euteiches*. The center of the diamond depicts the overall mean effect size, and the width reflects its confidence interval. CI = confidence interval.

In this meta-analysis, a natural log response ratio (*lnR*) value less than zero represents inhibition of *A. euteiches* growth and suppression of root rot symptoms*.* In contrast, a *lnR* value greater than zero shows no inhibition of the pathogen, no suppression of disease symptoms, and more severe disease. Thus, a value of zero suggests no treatment effect on *A. euteiches* and disease incidence. Our meta-analysis on cumulative efficacy detected a significant (*p* < .001) negative effect size [−0.411 (CI −0.516 to −0.306)] favoring inhibition of *A. euteiches* and suppression of disease symptoms (Figure 1).

The subgroups under the respective moderator variables were considered significantly different from each other and the overall mean when there was no confidence interval overlap. Also, the true difference, that is, heterogeneity among studies within each moderator variable, was determined based on ***I*** ^*2*^ and ***P***_***hetero***_. Results are grouped according to moderator variables as application method, biocontrol agent richness, biocontrol agent type, study type, and reporting system. A random-effects model is used to combine studies within each subgroup, and the subgroups were further combined using the same model. Then, the resulting overall effect size was used to determine the moderator variable’s impact on *A. euteiches* inhibition or suppression of disease symptoms.

#### 3.1.1 Method of Application

**FIGURE 2:**
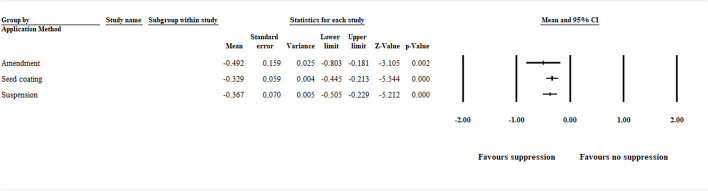
Effect of biocontrol agent application method on aphanomyces root rot suppression. The analysis detected a significant (*p* < .05) negative effect size for amendment, seed coating, and suspension. The center of the horizontal line depicts the effect size, and the width reflects its confidence interval. Number of studies: Amendment 35; Seed coating 79; Suspension 45. CI = confidence interval.

The analysis detected a significant (*p* < .05) negative effect size [−0.492 (CI −0.803 to −0.181)], favoring disease suppression when the application method was analyzed in relation to amendments (i.e., plant growing media amended with compost, green manure, and other plant products). Also, application as seed coating and liquid suspension showed a significant (*p* < .001) negative effect size [−0.329 (CI −0.445 to −0.213))] and [−0.367 (CI −0.505 to −0.229)], favoring disease suppression, respectively (Figure 2, Supplementary Figure S2 and Supplementary Table S3).

#### 3.1.2 Biocontrol Agent Richness

**FIGURE 3:**
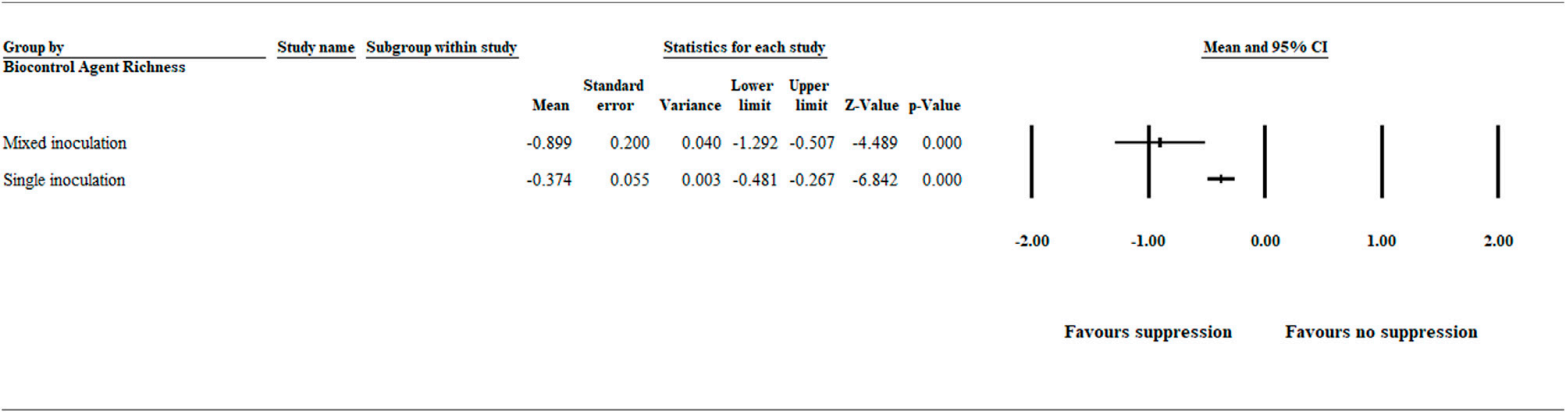
Effect of biocontrol agent richness on aphanomyces root rot suppression. The analysis detected a significant (*p* < .001) negative effect size for mixed and single organism inoculation favoring disease suppression. The center of the horizontal line depicts the effect size, and the width reflects its confidence interval. Number of studies: Mixed organism inoculation 11; Single organism inoculation 151. CI = confidence interval.

The analysis of the biocontrol agent richness effect on suppression of *A. euteiches* was categorized as single and mixed organism inoculation. The result showed a significant (*p* < .001) negative effect size [−0.899 (CI −1.292 to −0.507)] and [−0.374 (CI −0.481 to −0.267)] for mixed and single organism inoculation, respectively (Figure 3 and Supplementary Table S3).

#### 3.1.3 Biocontrol Agent Type

**FIGURE 4:**
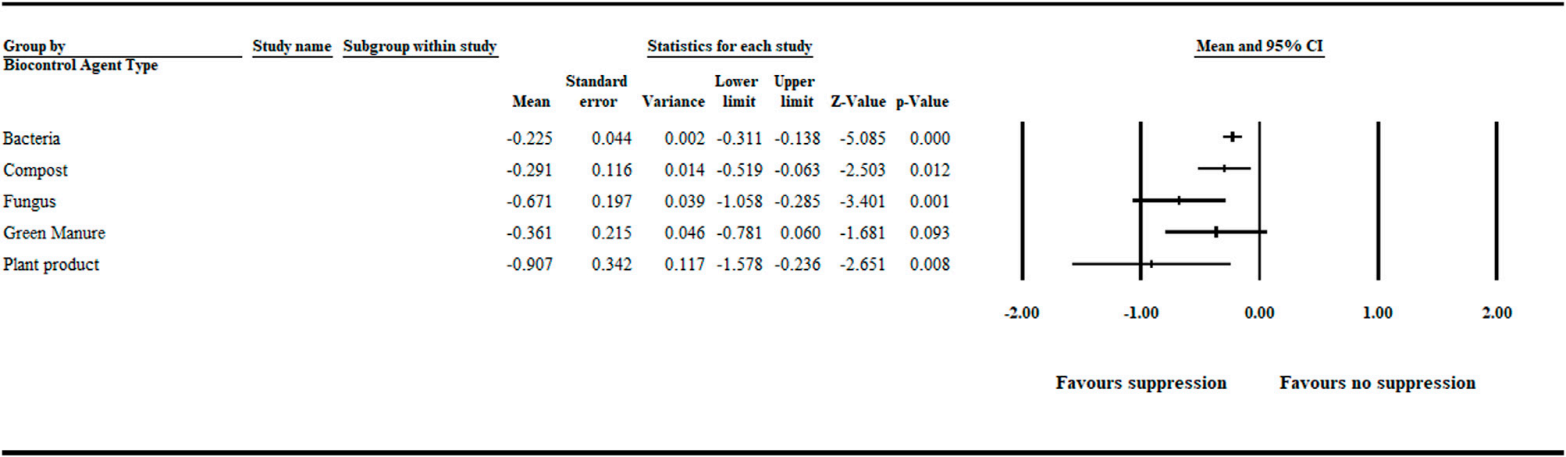
Effect of biocontrol agent type on aphanomyces root rot suppression. The analysis detected a significant (*p* < .05) negative effect size for bacterial, compost, fungal and plant product treatments. Number of studies: Bacteria 93; Compost 9; Fungus 26; Green manure 16; Plant product 12. The center of the horizontal line depicts the effect size, and the width reflects its confidence interval. CI = confidence interval.

In this meta-analysis, in addition to bacterial and fungal agents, other treatments, such as compost, green manure, and plant products (e.g., seed powder) used to suppress aphanomyces root rot were considered as biological treatments. Hence, bacteria, fungi, compost, green manure, and plant products were deemed individual levels in this category. The meta-analysis detected a significant negative effect size for bacteria [−0.225 (CI −0.311 to −0.138)], compost [−0.291 (CI −0.519 to −0.063)], fungi [−0.671 (CI −1.058 to −0.285)], and plant product [−0.907 (CI −1.578 to −0.236)] treatments. However, the summary effect size for green manure treatments was not significant (Figure 4 and Supplementary Table S3).

**FIGURE 5:**
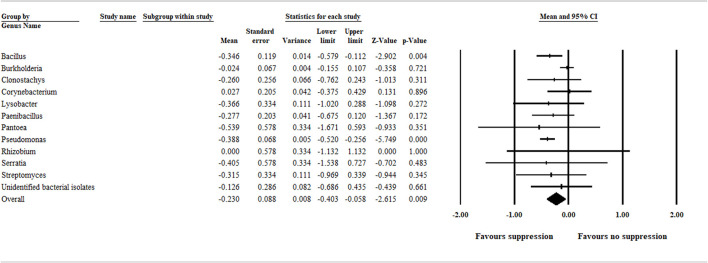
A separate meta-analysis on the bacterial treatment category indicates bacterial biocontrol agents within the genus *Bacillus* and *Pseudomonas* have a greater efficacy compared with others in the same category. The center of the diamond depicts the overall mean effect size, and the width reflects its confidence interval. CI = confidence interval. The *Pseudomonas cepacia* tested as a biocontrol agent in the Parke et al., 1991; and King and Parke, 1993 papers were included in the Burkholderia analysis, not in the *Pseudomonas* analysis.

Due to the relatively high number of treatments within the bacterial category, a separate analysis based on genus level grouping was performed to detect bacterial biocontrol agents (genus level) exhibiting greater efficacy compared with others in the same category. Our analysis detected bacterial biocontrol agents within the genus *Bacillus* and *Pseudomonas* as having a significant and greater efficacy in suppressing aphanomyces root rot (Figure 5).

#### 3.1.4 Study Type

**FIGURE 6:**
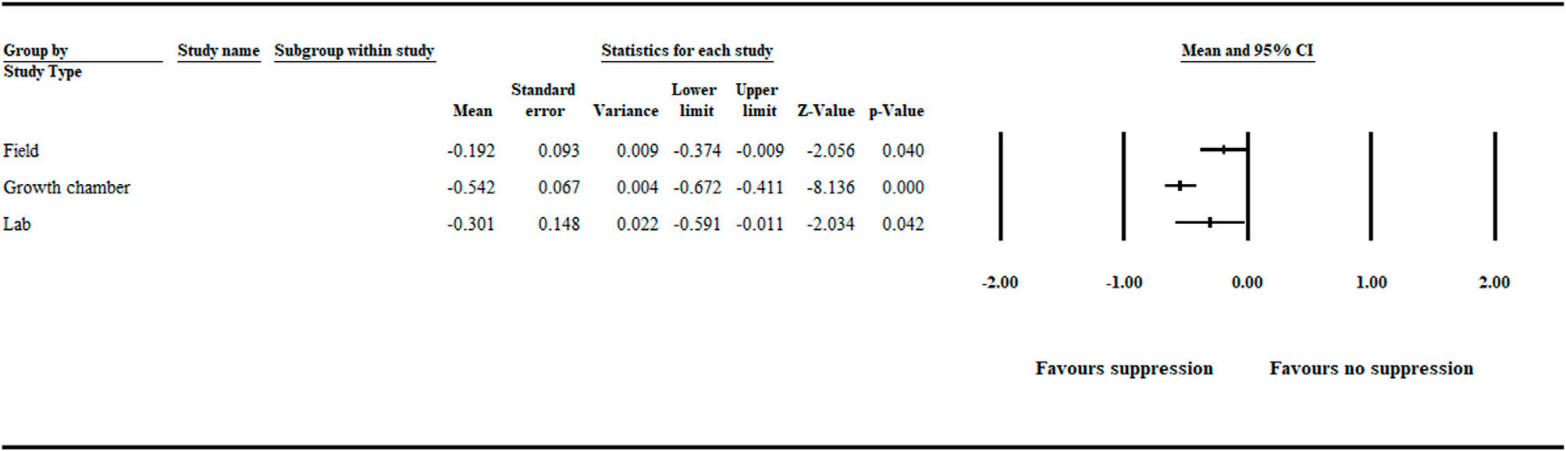
Study type impact on aphanomyces root rot suppression. The analysis detected a significant (*p* < .05) negative effect size for lab, growth chamber, and field studies. Number of studies: Lab 20; Growth chamber 101; Field 41. The center of the horizontal line depicts the effect size, and the width reflects its confidence interval. CI = confidence interval.

The analysis detected a significant (*p* < .05) negative effect size [−0.301 (CI −0.591 to −0.011)], [−0.542 (CI −0.672 to −0.411)] and [−0.192 (CI −0.374 to −0.009)], favoring disease suppression when study type was assessed in relation to lab, growth chamber, and field studies, respectively (Figure 6, Supplementary Figure S3 and Supplementary Table S3).

#### 3.1.5 Reporting System

**FIGURE 7:**
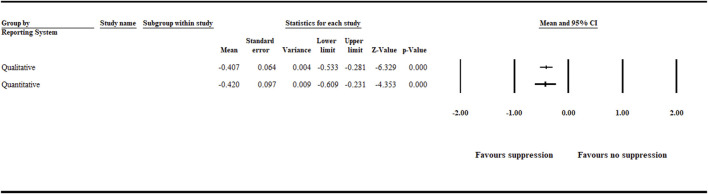
Effect of reporting system on aphanomyces root rot suppression. The analysis detected a significant (*p* < .001) negative effect size for the quantitative and qualitative reporting systems. Number of studies: Qualitative 111, and Quantitative 51. CI = confidence interval.

The analysis detected a significant (*p* < .001) negative effect size [−0.407 (CI −0.533 to −0.281)] and [−0.420 (CI −0.609 to −0.231)] for the quantitative and qualitative reporting systems (Figure 7 and Supplementary Table S3).

### 3.2 Test of Heterogeneity

The null hypothesis for heterogeneity is that the studies share a common effect size. The test of heterogeneity addresses whether the observed dispersion among effects exceeds the amount that would be expected by chance. As indicated in section 2.4, the Q statistic distributed as Chi-square shows the observed dispersion under the null hypothesis, and its anticipated value is equal to the degrees of freedom. Because Q tests the null hypothesis and assumes no dispersion across effect sizes, it is essential to quantify this dispersion. Hence, the dispersion is quantified based on the I^2^ value. Our heterogeneity analysis quantified that the overall I^2^ is greater than 84 for each effect variable, which means that more than 84% of the observed variance between studies is due to real differences in the effect size. Thus, less than 16% of the observed variance would be expected based on a random error (Table 1).

**TABLE 1:** Measures used to quantify dispersion across effect sizes in each moderator variable.

Moderator variable	Levels	Heterogeneity				
		N	Q-value	df (Q)	*p*-value	I-squared
Application method	Amendment	35	422	34	<.001	91.93
	Seed coating	79	618	78	<.001	87.37
	Suspension	45	21	44	.999	0.00
	Overall	159	1128	158	<.001	86.00
Biocontrol agent richness	Mixed inoculation	11	81	10	<.001	87.68
	Single inoculation	151	1165	150	<.001	87.13
	Overall	162	1291	161	<.001	87.53
Biocontrol agent type	Bacteria	93	365	92	<.001	74.81
	Compost	9	10	8	.283	17.95
	Fungus	26	108	25	<.001	76.81
	Green manure	16	221	15	<.001	93.21
	Plant product	12	130	11	<.001	91.52
	Overall	156	1018	155	<.001	84.77
Study type	Field	41	266	40	<.001	85.06
	Growth chamber	101	838	100	<.001	88.07
	Lab	20	26	19	.119	28.04
	Overall	162	1291	161	<.001	87.53
Reporting system	Qualitative	111	860	110	<.001	87.22
	Quantitative	51	421	50	<.001	88.13
	Overall	162	1290	161	<.001	87.53

A random-effects model was used to combine studies within each subgroup, and the same model was used to combine subgroups and yield the overall heterogeneity measures.

### 3.3 Publication Bias

**FIGURE 8:**
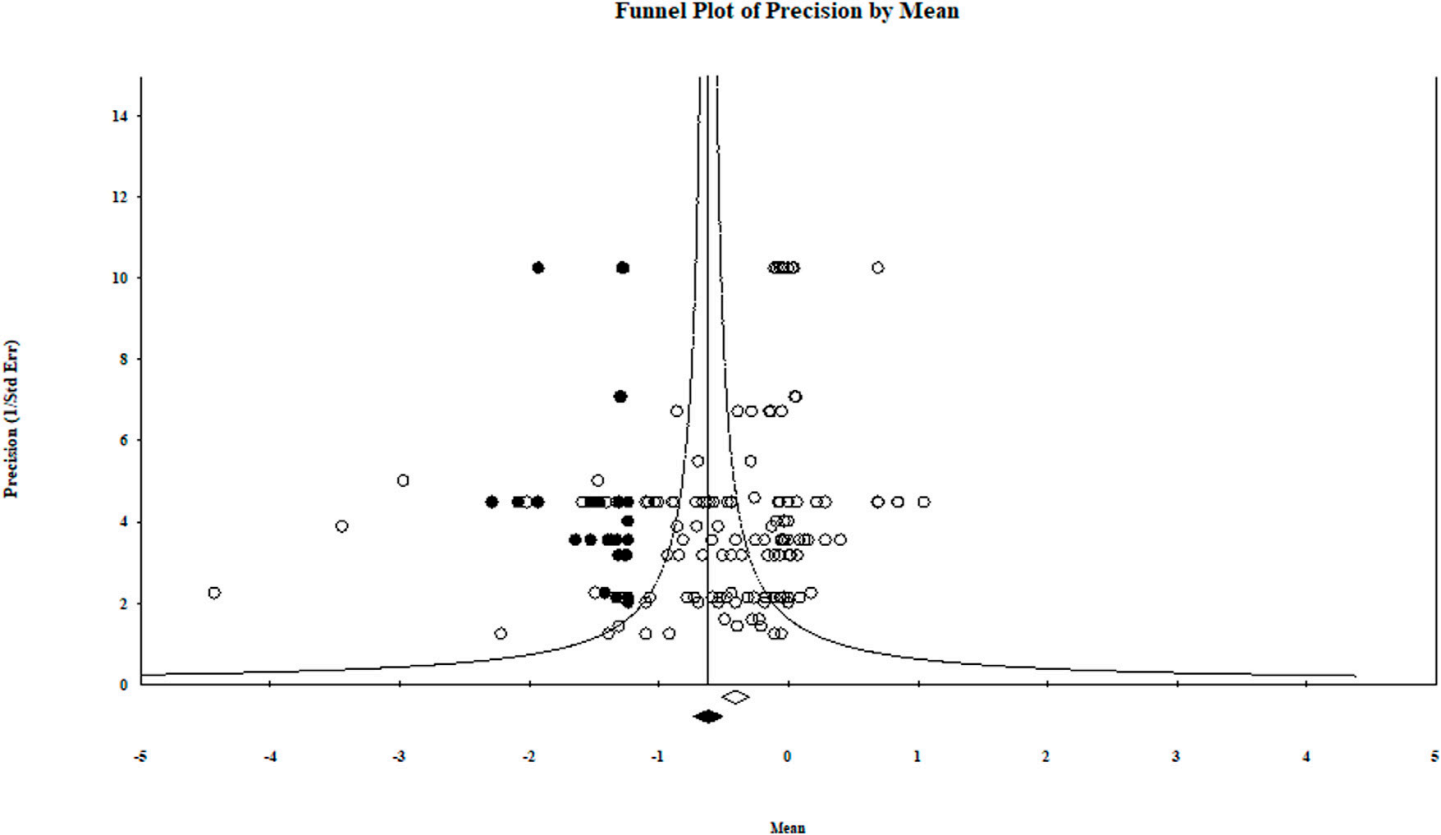
Trim-and-fill plot for log response ratio showing asymmetrical distribution of studies about the combined effect size or mean (i.e., the new adjusted effect size). This funnel plot is a measure of study size precision (standard error^−1^) on the vertical axis as a function of effect size on the horizontal axis. 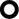
Observed studies; 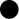
imputed studies; 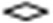
original effect size 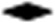
recomputed combined effect size. The program suggested 39 studies missing to the left of the mean. The centers of the diamonds depict the overall mean effect sizes, and the widths reflect the confidence interval.

Our comprehensive meta-analysis detected evidence of publication bias. Both large and small studies included in this meta-analysis did not have the expected variability around the overall effect size across the range of standard errors (precision) (Table 2). Also, the presence/absence of publication bias was checked by a graphical representation using a funnel plot (Figure 8). Within the Egger regression test, each summary effect had a *p*-value less than .05, indicating the presence of publication bias (i.e., there was a tendency for effect size to increase as study size decreased; Table 2). The Duval and Tweedie (2000) trim-and-fill method based on a random-effects model initially trimmed the most extreme small studies, looked for missing studies, and located the unbiased effect in an iterative procedure. The method then populated the plot by reinserting the trimmed studies until the funnel plot established symmetry on the adjusted (new) summary effect. Finally, the original studies were added back to the analysis along with their imputed counterparts to obtain an appropriate variance. In cases in which between-study heterogeneity exists (as it was in our meta-analysis) Duval and Tweedie’s trim and fill may incorrectly adjust for publication bias and result in a wrongly adjusted summary effect (Terrin et al., 2003). The issue associated with missing studies is that their absence in the analysis may lead to an exaggerated summary effect. In our meta-analysis, however, the new summary effect value adjusted for missing studies was further from the point of no impact (value = 0) than the biocontrol treatments’ original overall effect size value (Figure 8). Actually, if the new summary effect and suggested adjustments are legitimate for the biological control of aphanomyces root rot (i.e., the missing studies are valid), then Duval and Tweedie’s trim-and-fill analysis indicates an even more significant impact of the biocontrol agent treatments in suppressing aphanomyces root rot. Therefore, this publication bias must be acknowledged in the interpretation of the analyses.

**TABLE 2:** Variables used in characterizing publication bias for each moderator effect size.

Moderator variable	Summary effect^a^			Funnel plot^b^	Egger’s regression intercept^c^		Duval and tweedie trim and fill^d^	
	N	*lnR*	*P*	Yes	Intercept	*P*	Adjusted	No. impute
Application method	159	−0.36	<.001	Yes	−1.53	<.001	−0.57	37
Biocontrol agent richness	162	−0.41	<.001	Yes	−1.64	<.001	−0.62	39
Biocontrol agent type	156	−0.26	<.001	Yes	−1.41	<.001	−0.51	29
Study Type	162	−0.41	<.001	Yes	−1.64	<.001	−0.62	39
Reporting system	162	−0.41	<.001	Yes	−1.64	<.001	−0.62	39

a Summary effect: N, number of studies; *lnR*, natural log of overall summary effect; *P*, the probability that summary effect is 0.

b Funnel plot appears asymmetrical.

c Egger’s regression intercept: Intercept, this is a test for the Y-intercept = 0, *P* probability that the intercept is 0.

d Duval and Tweedie trim and fill: adjusted, new summary effect after imputing missing studies using an iterative trim and fill procedure. No. impute, number of studies imputed in the trim and fill analysis.

## 4 Discussion

Our meta-analysis provides strong evidence of an overall aphanomyces root rot suppression by biological control agents. These agents played an essential role in reducing aphanomyces root rot severity. The investigation also confirmed that the extent of disease suppression varied widely among experiments with the natural log response ratio ranging from –4.43 (i.e., strong disease suppression) to +1.05 (displaying no suppression relative to the control). Although the cumulative analysis effect size suggested disease suppression, this was not uniform across the various moderator variables and levels.

Once a potential biocontrol agent is identified, it is essential to understand the method of application that offers the highest efficacy. This is because, for effective biocontrol of soil-borne plant diseases, such as aphanomyces root rot, the proliferation of the biocontrol agent after introduction into the soil is one of the significant considerations. Therefore, an efficient application method needs to promote the biocontrol agent’s rhizosphere competence, which includes producing an inoculum that survives, grows, and colonizes the rhizosphere and the plant roots over a considerable period (Yigit and Dikilitas, 2007; Compant et al., 2005; El-Mougy and Abdel-Kader, 2019). In this regard, our analysis indicates that the different types of application methods significantly influence aphanomyces root rot suppression. For example, significant disease suppression was obtained when biological treatments were applied as a seed coating and liquid suspension.

Several biocontrol agents were reported to be effective when applied as a seed coating and liquid suspension. For example, Abdel-Kader et al. (2012) investigated different application methods of biocontrol agents for controlling oomycete and fungal root rot incidence of some vegetables under greenhouse conditions. Their results indicate that, although seed coating application was significant in reducing root rot incidence, soil drenched with different bioagents showed more efficacy against root rot in cucumber, cantaloupe, tomato, and pepper in the postemergence growth stage. Furthermore, a review study by Rocha et al. (2019) describes seed coating as an essential tool for delivering beneficial microbes to agricultural crops to promote crop growth, yield, and crop protection against pathogens. Therefore, as detected in our meta-analysis, liquid suspension and seed coating application are efficient methods to obtain a significant level of aphanomyces root rot suppression. Moreover, choosing the method of application that offers the highest efficacy relies on a better understanding of the biological and application-oriented factors influencing the disease suppression potential of the biocontrol agent (Ojiambo and Scherm 2006).

We investigated the impact of single and mixed organism inoculation of biocontrol agents on aphanomyces root rot suppression. Although it is challenging to draw a strong common conclusion due to the small sample size (*n* = 11), the analysis indicates that mixed organism inoculation had greater disease suppression efficacy than single organism inoculation. Such a phenomenon can be attributed to the notion that mixed inoculation of biocontrol agents can provide a more significant disease suppression effect than a single organism inoculation due to synergistic or additive effects. This finding is consistent with studies by Stiling and Cornelissen (2005) and Chandrasekaran et al. (2016), who conducted a meta-analysis review on biological control agents’ performance and the genus *Pseudomonas* as a biological control agent against bacterial wilt, respectively. However, our results contradict a study finding by Dandurand and Knudsen (1993) in which they report aphanomyces root rot suppression was not significantly different when pea seeds were treated with a combination of *T. harzianum* and *Pseudomonas fluorescens* strains compared with treatment with *T. harzianum* alone. Furthermore, among the biocontrol agent types, compost, plant product, fungal, and bacterial biocontrol agents were effective biocontrol treatments for suppressing aphanomyces root rot.

In our meta-analysis study, multiple biocontrol agents studied in an article were treated as independent studies, and each represented individual units. Therefore, owing to the relatively high total number of bacterial biocontrol agents (*n* = 93) evaluated for biocontrol efficacy, it is possible that a bacterial biocontrol agent with high efficacy could be hidden in such groupings. Therefore, care needs to be given when interpreting grouping results. In this regard, our separate analysis on the bacterial biocontrol agents at a genus level grouping detected bacterial strains in the genus *Bacillus* and *Pseudomonas* significantly favoring disease suppression (Figure 5). This finding is consistent with those of Wakelin et al. (2002), who investigated *A. euteiches* growth inhibition and suppression of pea root rot using spore-forming bacteria, including *Bacillus* species. Similarly, several *Pseudomonas* species that exhibited biocontrol efficacy against aphanomyces root rot and other oomycete diseases*,* such as Pythium damping off, were reported (Parke et al., 1991; King and Parke, 1993; Reddy, 2002).

We observed that disease suppression was favored in both growth chamber and field studies, indicating the potential for biological control of aphanomyces root rot. However, more significant suppression was achieved in growth chamber trials (Figure 6). Others similarly indicate that biological control agents produce better consistency and higher efficacy against various plant pathogens under controlled growth chamber conditions than field trials (Guetsky et al., 2001; Mark et al., 2006; Nicot, 2011). For the most part, the irregularity in biocontrol efficacy in the field could be attributed to various factors, including soil type and condition; climatic variations, such as temperature and humidity; and UV irradiation encountered in field conditions. Another reason could be the lack of ecological competence that reduces the survival and colonization ability of the biocontrol agents. Also, inconsistent production of bioactive metabolites required to suppress the pathogen and inadequate formulation and application methods can contribute to inconsistent biocontrol efficacy (Elad and Stewart, 2007; Mark et al., 2006; Ruocco et al., 2011). Moreover, under field conditions, usually more than one pathogen is part of a complex that causes the disease to a crop. For example, aphanomyces root rot often occurs in a complex with other root rot–causing pathogens (Parke et al., 1991; Xue 2003; Hughes and Grau 2013). Finally, maintaining the population of biocontrol agents above a certain level in the soil is an essential factor that affects biocontrol efficacy in both growth chamber and field conditions (Yuan et al., 2014). Because growth chamber studies offer a better opportunity to control experimental conditions, maintaining the population of biocontrol agents above a certain level is more feasible in growth chamber studies.

One of the challenges in plant pathology studies is to develop standardized qualitative and quantitative disease incidence and severity measures that integrate numerical and observational data. Another challenge is the severity of the disease in relation to a biocontrol agent’s capability to control it. Biocontrol agents have their limitations in terms of the severity of the disease that they can control. Furthermore, moving from a controlled environment to the field, a biocontrol agent will very likely encounter populations of the target pathogen that are genetically different with different virulence than the populations to which it was exposed during screening procedures. In our meta-analysis, between data entries used to analyze the impact of reporting systems, both qualitative and quantitative reporting systems favored the detection of aphanomyces root rot suppression. Qualitative reporting systems, such as the “disease rating scale,” are more common in plant pathological studies, such as biological control of aphanomyces root rot. However, it is prone to bias compared with quantitative reporting systems due to its subjective nature and lack of standardized measuring tools. Therefore, to increase precision and minimize error, there is a need to establish an agreed-upon standardized system for assessing biocontrol agent performance that integrates direct and indirect quantitative disease incidence measuring reports, for example, quantifying pathogen infestation levels and measuring plant health and growth monitoring parameters such as plant dry weight and plant height.

## 5 Conclusion

Our meta-analysis detected factors such as biocontrol application method, biocontrol agent type and richness, study type, and reporting system as affecting the measured efficacy of biological control of aphanomyces root rot. In addition, some of the findings strengthened the prevailing view that most biocontrol agents display higher efficacy under controlled plant growing conditions than field trials. Also, strains within the genera *Bacillus* and *Pseudomonas* favored more significant suppression among bacterial biocontrol agents. Moreover, biocontrol of aphanomyces root rot was significantly suppressed when an inoculant consisting of mixed organisms was used compared with one biocontrol agent alone. Therefore, our analysis demonstrates there is very good potential for biological control of aphanomyces root rot.

Initially, we also aimed to include aphanomyces biocontrol mechanism and plant type as moderator variables; however, these two potential categorical variables were not included due to insufficient data. Therefore, future studies to elucidate the mechanism(s) of biological control of aphanomyces root rot need to be a priority. These include understanding the biology of the biocontrol agents and their natural fitness that play a crucial role in colonizing and successfully establishing in soils conducive to *A. euteiches*, including warm (23°C) moist soil conditions. Also, identifying a biocontrol agent that naturally forms a symbiotic association with pea plants (for example, *Rhizobium* spp) could play an essential role in clearly determining the “best and most effective” biocontrol agents for aphanomyces root rot. Moreover, identification and characterization of the mechanistic nature of disease suppression offer an additional insight into whether it is beneficial to utilize an active metabolite to control aphanomyces root rot than the biocontrol agent itself.

## Supplementary Material

Supplementary Figure S1 Diagrammatic representation of the steps involved in selecting articles.

Supplementary Figure S2 Application method effect size meta-regression impacting aphanomyces root rot suppression. Error bars indicate confidence interval (*p* < .05). Number of studies: Amendment 35; Seedcoat 79; and Suspension 45.

Supplementary Figure S3 Study type effect size meta-regression impacting aphanomyces root rot suppression. Error bars indicate confidence interval (*p* < .05). Number of studies: Field trial 41, Growth chamber 101 and Lab assay 20.
