# Supplementary figures and images for: A Meta-Analysis to Determine the State of Biological Control of Aphanomyces Root Rot
Source: Front Mol Biosci. 2022 Feb 2;8:777042. doi: 10.3389/fmolb.2021.777042 (PMC8847612; doi:10.3389/fmolb.2021.777042)

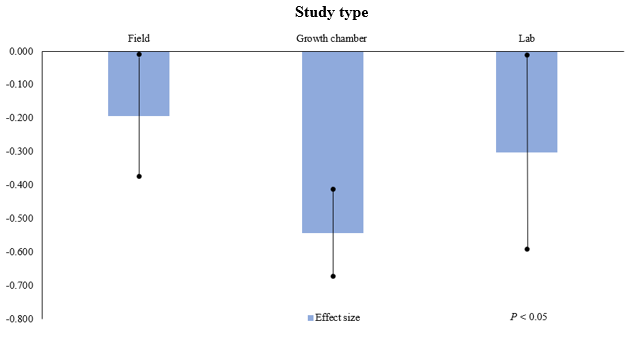

Supplement: Supplementary file 2 [file Image3.tif]

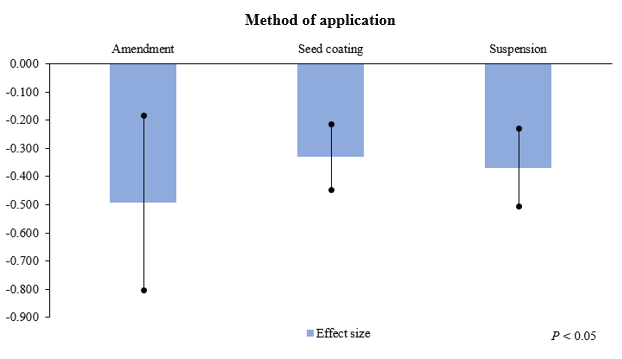

Supplement: Supplementary file 3 [file Image2.tif]

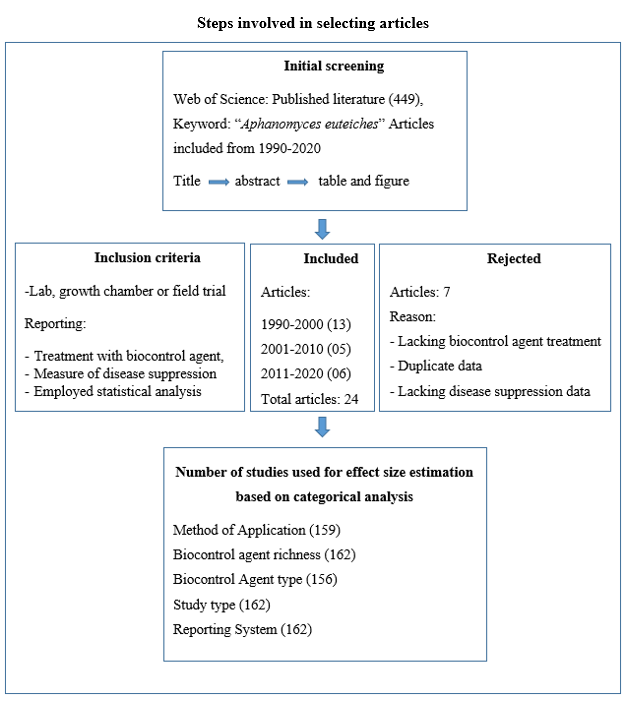

Supplement: Supplementary file 4 [file Image1.tif]
