# Supplementary material for: A Meta-Analysis to Determine the State of Biological Control of Aphanomyces Root Rot
Source: Front Mol Biosci. 2022 Feb 2;8:777042. doi: 10.3389/fmolb.2021.777042 (PMC8847612; doi:10.3389/fmolb.2021.777042)
Supplement: Supplementary file 5 [file Table3.docx]

**Table S3.** Application method, biocontrol agent richness, biocontrol gent type, study type and reporting system effect on aphanomyces root rot suppression.

| Moderator variable | Levels | Effect size and 95% confidence interval | | | | | | |
| --- | --- | --- | --- | --- | --- | --- | --- | --- |
|  |  | N | lnR | Standard error | Variance | Lower limit | Upper limit | *P*-value |
| Application method | Amendment | 35 | -0.492 | 0.159 | 0.025 | -0.803 | -0.181 | 0.002 |
|  | Seedcoat | 79 | -0.329 | 0.059 | 0.004 | -0.445 | -0.213 | <0.000 |
|  | Suspension | 45 | -0.367 | 0.070 | 0.005 | -0.505 | -0.229 | <0.000 |
|  | Overall | 159 | -0.356 | 0.044 | 0.002 | -0.442 | -0.271 | <0.000 |
| Biocontrol agent richness | Mixed inoculation | 11 | -0.899 | 0.200 | 0.040 | -1.292 | -0.507 | <0.000 |
|  | Single inoculation | 151 | -0.374 | 0.055 | 0.003 | -0.481 | -0.267 | <0.000 |
|  | Overall | 162 | -0.411 | 0.053 | 0.003 | -0.514 | -0.307 | <0.000 |
| Biocontrol agent type | Bacteria | 93 | -0.225 | 0.044 | 0.002 | -0.311 | -0.138 | <0.000 |
|  | Compost | 9 | -0.291 | 0.116 | 0.014 | -0.519 | -0.063 | 0.012 |
|  | Fungus | 26 | -0.671 | 0.197 | 0.039 | -1.058 | -0.285 | 0.001 |
|  | Green Manure | 16 | -0.361 | 0.215 | 0.046 | -0.781 | 0.060 | 0.093 |
|  | Plant product | 12 | -0.907 | 0.342 | 0.117 | -1.578 | -0.236 | 0.008 |
|  | Overall | 156 | -0.264 | 0.039 | 0.002 | -0.341 | -0.187 | <0.000 |
| Study type | Field | 41 | -0.192 | 0.093 | 0.009 | -0.374 | -0.009 | 0.040 |
|  | Growth chamber | 101 | -0.542 | 0.067 | 0.004 | -0.672 | -0.411 | <0.000 |
|  | Lab | 20 | -0.301 | 0.148 | 0.022 | -0.591 | -0.011 | 0.042 |
|  | Overall | 162 | -0.411 | 0.051 | 0.003 | -0.509 | -0.309 | <0.000 |
| Reporting system | Qualitative | 111 | -0.407 | 0.064 | 0.004 | -0.533 | -0.281 | <0.000 |
|  | Quantitative | 51 | -0.420 | 0.097 | 0.009 | -0.609 | -0.231 | <0.000 |
|  | Overall | 162 | -0.411 | 0.053 | 0.003 | -0.516 | -0.306 | <0.000 |

A random-effects model was used to combine studies within each subgroup, and the same model was used to combine subgroups and yield the overall effect size.
